# Optimized nerve block techniques while performing percutaneous hepatic ablation: **Literature review and practical use**

**DOI:** 10.1016/j.jimed.2020.06.002

**Published:** 2020-08-12

**Authors:** DM Liu, A Hadjivassiliou, D Valenti, SG Ho, D Klass, JB Chung, PT Kim, LM Boucher

**Affiliations:** a Associate Professor, Faculty of Medicine, University of British Columbia, Canada; b Voluntary Professor, Miller School of Medicine, University of Miami, USA; c Department of Radiology - Division of Interventional Radiology, Vancouver General Hospital, Canada; d Department of Radiology, Vancouver General Hospital, Canada; e Department of Surgery Division of Hepatopancraticobiliary Surgery/Liver Transplantation, Vancouver General Hospital, Vancouver, Canada; f Assistant Professor, Faculty of Medicine, McGill University, Department of Radiology, Division of Interventional Radiology, McGill University Health Centre, Montreal, Canada; g Associate Professor, Faculty of Applied Science, University of British Columbia, Canada; h Clinical Professor, Faculty of Medicine, University of British Columbia, Canada; i Clinical Associate Professor, Faculty of Medicine, University of British Columbia, Canada

## Abstract

Percutaneous image guided thermal ablation has become a cornerstone of therapy for patients with oligometastatic disease and primary liver malignancies. Evolving from percutaneous ethanol injection (PEI), thermal ablation utilizing radiofrequency ablation (RFA) and microwave ablation (MWA) have become the standard approach in the treatment of isolated lesions that fit within the size criteria for curative intent therapy (typically 3-4cm). With the evolution of more intense thermal ablation, such as MWA, the dramatic increase in both the size of ablation zone and intensity of heat generation have extended the limits of this technique. As a result of these innovations, intra-procedural and post-procedural pain have also significantly increased, requiring either higher levels of intravenous sedation or, in some institutions, general anesthesia.

In addition to the increase in therapeutic intensity, the use of intravenous sedation during aggressive ablation procedures carries the risk of over-sedation when the noxious insult (i.e. the ablation) is removed, adding further difficulty to post-procedural recovery and management. Furthermore, high subdiaphragmatic lesions become challenging in this setting due to issues relating to sedation and compliance with breath hold/breathing instructions. Although general anesthesia may mitigate these complications, the added resources associated with providing general anesthesia during ablation is not cost effective and may result in substantial delays in treatment. The reduction of Aerosol Generating Medical Procedures (AGMP), such as intubation due to the COVID-19 Pandemic, must also be taken into consideration. Due to the potential increased risk of infection transmission, alternatives to general anesthesia should be considered when safe and possible.

Upper abdominal regional nerve block techniques have been used to manage pain related to trauma, surgery, and cancer; however, blocks of this nature are not well described in the interventional radiology literature. The McGill University group has developed experience in using such blocks as splanchnic, celiac and hepatic hilar nerve blocks to provide peri-procedural pain control [1]. Since incorporating these techniques (along with hydrodissection with tumescent anesthesia), we have also observed in our high volume ablation center a dramatic decrease in the amount of sedatives administered during the procedure, a decrease in patient discomfort during localization and ablation, as well as decreased pain post-procedure. Faster time to discharge and overall reduction in room procedural time serve as added benefits.

The purpose of this publication is to outline and illustrate the practical application and use of nerve block/regional anesthesia techniques with respect to percutaneous hepatic thermal ablation.

## Introduction

**Fig. 1:**
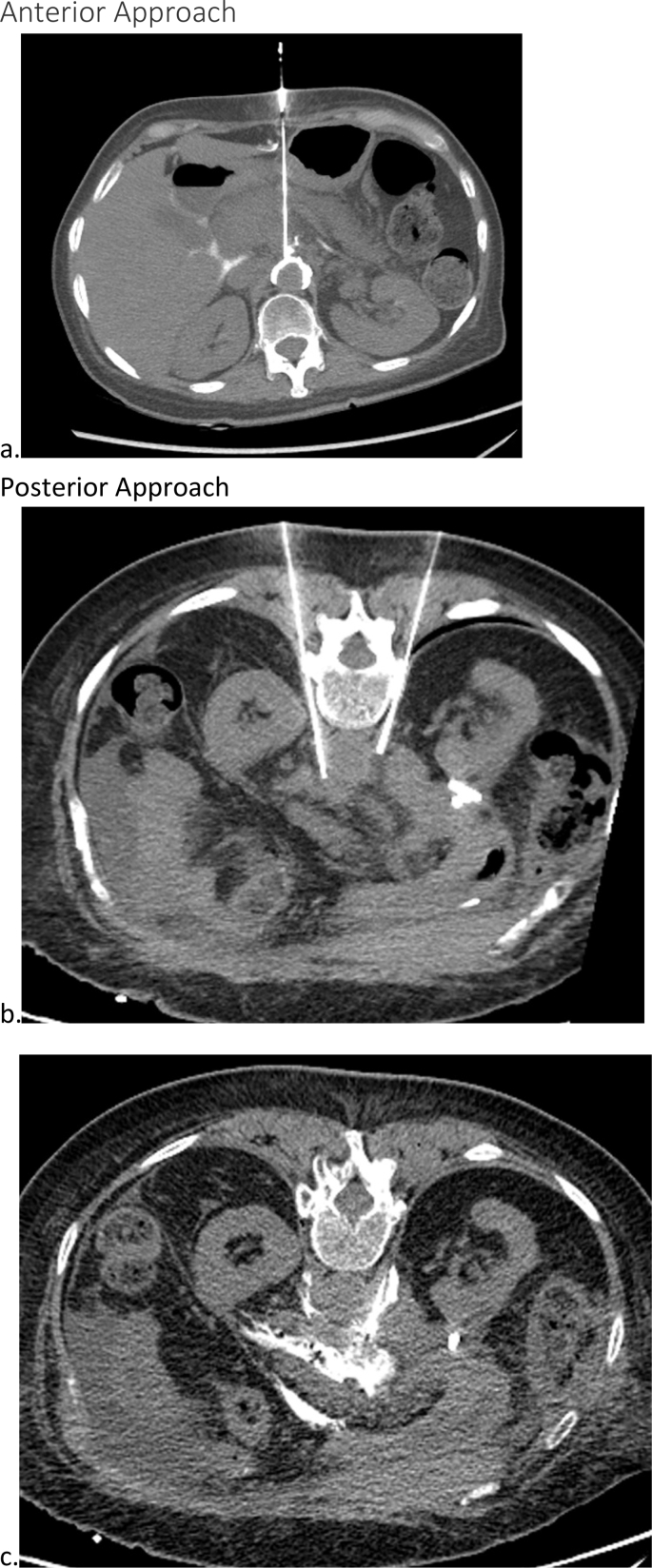
Celiac Block Technique Under CT Guidance (Anterior and Posterior approaches) Celiac plexus nerve block under CT guidance (anterior and posterior approach). CT guided transgastric approach to celiac axis using a 22 ​g needle, confirmed with dilute contrast injection distributing along the celiac axis and adjacent tissue (a). Transhepatic approaches may also be performed safely. CT guided posterior approach to celiac plexus, bilateral approach, using a paraspinal technique with confirmation of distribution via injection of dilute contrast (b+c). A single needle approach may be utilized, provided adequate distribution of contrast is achieved in the region of the celiac axis.

**Fig. 2:**
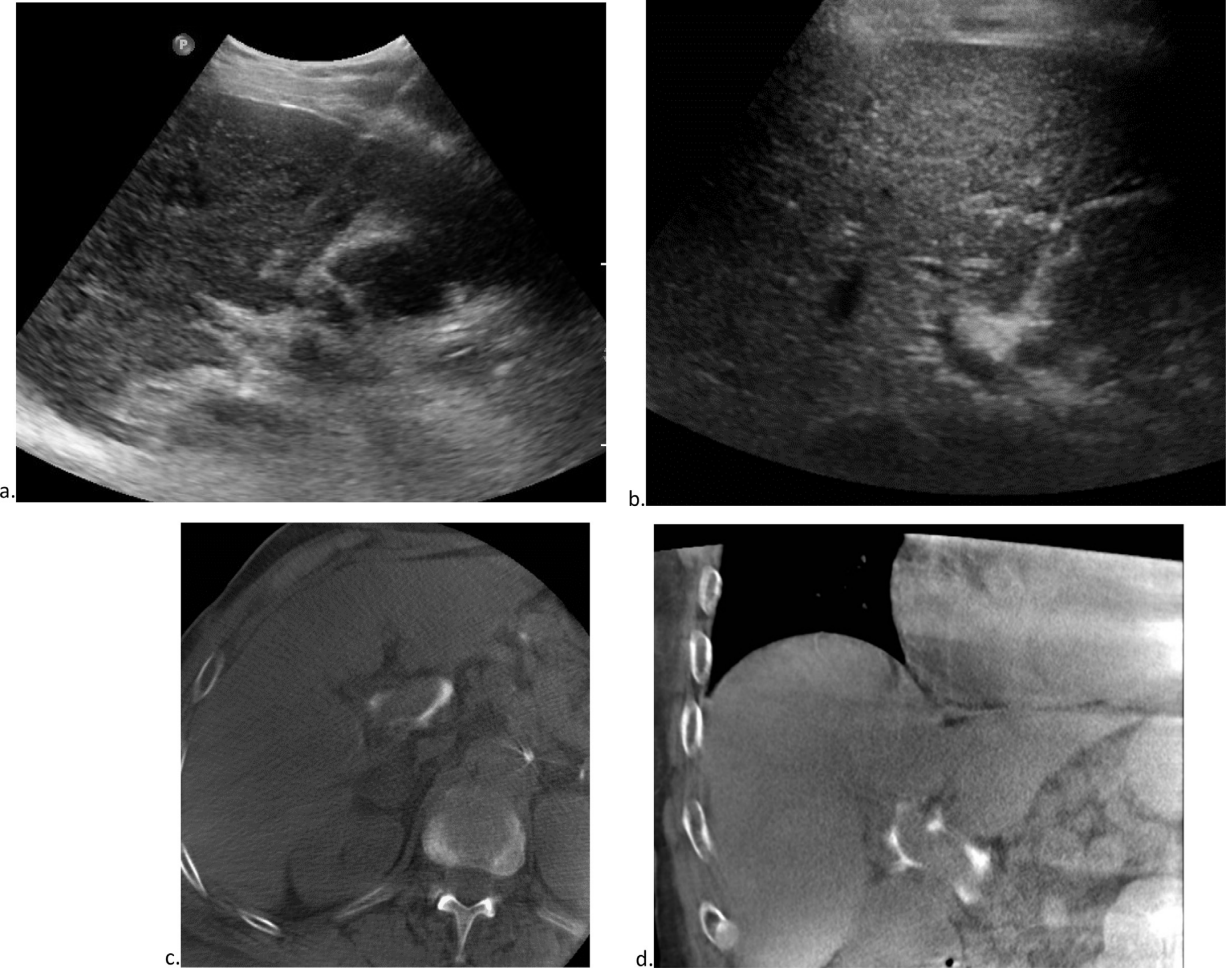
Perihilar Block Technique Under Ultrasound Guidance confirmed with Cone Beam CT Perihilar nerve block under ultrasound guidance requires appropriate visualization of the main or lobar portal vein in order to ensure a safe trajectory. Off axial images demonstrating needle trajectory towards the portal vein, with injection of local anesthetic in the target region (a + b). During injection, fluid around the portal vein may be visualized and a mild amount of resistance to the infusion may be encountered. Illustrative coronal Cone beam CT imaging of the distribution of infusate (with dilute contrast) demonstrates the distribution of local anesthetic along the branch and main portal vein (c). Ultrasound confirmation is usually all that is required in the clinical setting.

Percutaneous image guided thermal ablation has become a cornerstone of therapy for patients with oligometastatic disease and primary liver malignancies. Evolving from percutaneous ethanol injection (PEI), thermal ablation utilizing radiofrequency ablation (RFA) and microwave ablation (MWA) have become the standard approach in the treatment of isolated lesions that fit within the size criteria for curative intent therapy (typically 3–4 ​cm). With the evolution of more intense thermal ablation, such as MWA, the dramatic increase in both the size of ablation zone and intensity of heat generation have extended the limits of this technique. As a result of these innovations, intra-procedural and post-procedural pain have also significantly increased, requiring either higher levels of intravenous sedation or, in some institutions, general anesthesia. (see Fig. 1, Fig. 2)

In addition to the increase in therapeutic intensity, the use of intravenous sedation during aggressive ablation procedures carries the risk of over-sedation when the noxious insult (i.e. the ablation) is removed, adding further difficulty to post-procedural recovery and management. Furthermore, high subdiaphragmatic lesions become challenging in this setting due to issues relating to sedation and compliance with breath hold/breathing instructions. Although general anesthesia may mitigate these complications, the added resources associated with providing general anesthesia during ablation is not cost effective and may result in substantial delays in treatment. The reduction of Aerosol Generating Medical Procedures (AGMP), such as intubation due to the COVID-19 Pandemic, must also be taken into consideration. Due to the potential increased risk of infection transmission, alternatives to general anesthesia should be considered when safe and possible.

Upper abdominal regional nerve block techniques have been used to manage pain related to trauma, surgery, and cancer; however, blocks of this nature are not well described in the interventional radiology literature. The McGill University group has developed experience in using such blocks as splanchnic, celiac and hepatic hilar nerve blocks to provide peri-procedural pain control.^1^ Since incorporating these techniques (along with hydrodissection with tumescent anesthesia), we have also observed in our high volume ablation center a dramatic decrease in the amount of sedatives administered during the procedure, a decrease in patient discomfort during localization and ablation, as well as decreased pain post-procedure. Faster time to discharge and overall reduction in room procedural time serve as added benefits.

The purpose of this publication is to outline and illustrate the practical application and use of nerve block/regional anesthesia techniques with respect to percutaneous hepatic thermal ablation.

## Anatomy

Embryologically, the liver develops from the foregut endodermal epithelium. In the fetal period, it initially serves primarily as a poorly innervated hematopoietic organ. With time, multiple liver metabolic functions develop that necessitate accurate modulation, including via careful neural control.^2^ The hepatic neural signals are mediated by sympathetic and parasympathetic fibers. Sympathetic fibers originate from the T7-12 spinal thoracic levels and travel from the spine to the abdomen via the splanchnic nerves. The splanchnic nerves course on both sides of the anterior aspects of the lower thoracic vertebral bodies to the T11-12 level where they extend anteriorly to pierce the diaphragm.^3^ Upon entering the abdomen, the splanchnic nerves travel to the celiac ganglia, located on either side of the aorta at the celiac trunk level, anterior to the crux of the diaphragm medial to the adrenal glands.^4^ The parasympathetic nerves travel from the brainstem to the celiac plexus via the anterior (left) and posterior (right) vagus branches. The sympathetic and parasympathetic fibers, as well as some fibers from mixed nerves like the phrenic nerve, join together at the celiac plexus along the anterior surface of the aorta, inferior to the celiac artery origin, to form plexuses extending to the various organs of the upper abdomen.^5^

The hepatic nervous supply is formed by two plexuses. Nerves from the left portion of the celiac plexus and the parasympathetic branches from the posterior abdominal branch of the vagus connect to the liver via an anterior hepatic plexus that runs along the hepatic artery. The nerves from the right aspect of the celiac plexus travel in the posterior hepatic plexus along the portal vein^6^.

The sympathetic nerves are considered to be the primary carriers of noxious stimuli from the liver to the central nervous system.^7^ In the hepatic hilum, nerves course along the hepatic arterial, portal venous, and biliary systems. The hepatic hilar plate is a space in which the portal vein, hepatic artery, and biliary system divide into left and right lobar branches.^8^ This region is a potential area in which an injectate, such as a nerve blocking agent, could affect nerves surrounding all three elements, and is the target of the hepatic hilar block, a novel peripheral nerve block that is specific to the liver (see below).

## Technique

For any nerve block, a few basic principles should be followed to avoid complications:

1. For most blocks, a 21 or 22 ​g spinal or Chiba needle or smallest diameter needle should be used. Needles with echogenic tips can help facilitate ultrasound guided procedures.
2. Vital sign and EKG monitoring should be performed throughout the procedure to detect and manage potential autonomic nervous system derangement.
3. Optimal imaging technique should be used to visualize the targeted compartment. While CT or fluoroscopy might be necessary for deep blocks, ultrasound guidance provides real-time imaging that may be faster, safer, and potentially more accurate.
4. Confirmation of the extravascular location of the needle tip is essential. Confirmation techniques include: a) lack of blood aspiration, b) lack of epinephrine induced tachycardia upon injecting 3 ​cc of lidocaine with epinephrine, c) contrast injection demonstrating correct spread of contrast and no vascular uptake or d) injection of saline/D5W under ultrasound to confirm compartmentalization of injectate/infusate.
5. Various combinations of nerve block agents can be used from shorter-acting agents such as lidocaine (1–2 ​h) with safer profiles, to longer-acting agents such as bupivacaine or ropivacaine (6–8 ​h). The maximal weight-based dose should not be exceeded (lidocaine 5 ​mg/kg, bupivacaine 2 ​mg/kg, ropivacaine 3 ​mg/kg).^9^
6. A management plan, including access to intravenous lipid infusion for ‘lipid rescue’ technique should be available in case of intravascular toxicity.^10^^,^^11^
7. Careful post-procedural monitoring of the patient in the recovery room will be needed for 1–2 ​h, to ensure absence of delayed nerve block toxicity characterized by perioral numbness, light headedness, dizziness, visual/auditory disturbances, disorientation, seizures, or cardiac arrhythmias.

Effective blockage of noxious stimuli from the liver can be obtained by performing a hepatic hilar nerve block. For lesions that are close to the capsule, an added tumescent infiltration of the liver capsule or hydrodissection/artificial ascites mixed with anesthetic near the lesion may warrant consideration. For lesions close to the diaphragm, an added right phrenic nerve block may be necessary (taking into consideration the possibility of paralyzing the diaphragm, resulting in a high position of the liver.^12^ If a hepatic hilar nerve block is not possible, such as if the hilum is occupied by a mass, a celiac plexus block or splanchnic block may warrant consideration. Brief descriptions of these techniques are described below. Readers are encouraged to review source reference publications for further guidance.

### Hepatic hilar block

The target position of the needle tip for the hepatic hilar block is in the periportal fat near the bifurcation of the main portal vein into right and left portal branches. A trans-hepatic approach using ultrasound is favored. An approach through the left lobe is usually easier due to shorter distance to the target and absence of intervening ribs, but a trans-right hepatic lobe approach can also be used. Once the needle tip has been advanced into the periportal fat, confirmation of extravascular position is performed. Upon confirmation of needle position, 10–15 ​ml or 0.25% bupivacaine or 0.5% ropivacaine is injected. Although no specific complications have been seen so far, there is the potential risk of vascular injury to the liver.

### Celiac plexus block

The target position of the needle tip is anterior to the aorta between the origin of the celiac trunk and superior mesenteric artery. In thin patients, an anterior ultrasound approach can be used. Otherwise, a CT or fluoro-CT guided approach can be used. If an anterior approach is not possible, a posterior bilateral approach can be used targeting both celiac ganglia. Alternatively, a posterior unilateral trans-aortic or wide posterolateral unilateral approach attempting to place the needle as close as possible to the anterior wall of the aorta can be used. When the needle gets close to the aorta and the pulsations are strongly felt on the needle, it should no longer be advanced and final position confirmed. Once the needle tip is in position and absence of intravascular leakage is demonstrated, 10–15 ​ml of 0.25% bupivacaine or 0.5% ropivacaine is injected. Celiac block specific risks include transient diarrhea (44%) and postural hypotension (10–52%). Serious complications occur in <2% and include vascular and organ injury that are approach specific. An anterior approach can lead to GI or pancreatic injury. A posterior approach can lead to vascular injury including to spinal vessels with very rare complication of paralysis.^13^

### Splanchnic nerve block

The target position is along the anterior third of the T11 vertebral body on either side. A posterior fluoroscopic guided approach is most often used. The vertebral body is “squared” by angulating the fluoroscopy tube cranio-caudally. The tube is then angulated ipsilaterally until the concave contour of the side of the vertebral body is seen. Using a “down-the-barrel” approach, the needle is advanced alongside the vertebral body until its tip is at the anterior 1/3 of the vertebral body on the lateral view. The same procedure is performed on the opposite side. Once in position, with no evidence of intravascular leakage, 8 ​cc of 0.25% bupivacaine or 0.5% ropivacaine is injected on either side. Splanchnic nerve block risks include transient diarrhea (14–30%) and orthostatic hypotension (19–53%). Very rare complications include paralysis, sexual dysfunction, pneumothorax and chylothorax.^14^

### Hydrodissection with dilute local anesthetic (modified tumescent anesthesia technique)

The admixture of dilute forms of local anesthetic administered in the peritoneal space or intrabdominal compartment can be performed easily and without additional discomfort. Through dilution techniques first developed for plastic surgery procedures such as liposuction, buffered or dilute local anesthetic administered into the peritoneal cavity can provide simultaneous anesthesia to both peritoneal and visceral surfaces, thereby minimizing pain associated with peritoneal puncture, liver capsule puncture, and irritation due to thermal flux. Techniques to elicit iatrogenic ascites have been well described in the literature^15^, ^16^, ^17^; in brief, use of a modified Seldinger technique using a micropuncture access point (via ultrasound, and/or loss of resistance technique) allows for serial dilation and upsizing to peritoneal drain placement, thus allowing for larger volume ascites formation with dilute local anesthetic following localization and placement of an ablation needle/probe. Although concerns have been expressed relating to performing percutaneous ablation in the presence of ascites, the creation of iatrogenic ascites does not reflect hepatic function derangement and may be aspirated or removed at the conclusion of the procedure.

### Intercostal nerve block/paravertebral nerve block

Thoracic paravertebral block (TPVB), is a well-established technique intended to anesthetize the spinal nerve roots and sympathetic chain in the paravertebral space and may provide unilateral chest and abdominal wall analgesia. The block has been used to control pain specifically of hepatic origin from tumor infiltration,^18^ during percutaneous transhepatic biliary drainage^19^^,^^20^ or following liver trauma.^21^ Studies utilizing paravertebral and intercostal nerve block techniques have confirmed its value in decreasing narcotic requirements, decreasing pain prior, during, and following ablation, as well as increasing operator satisfaction.

### Intraarterial INFUSION during combined intraarterial and ablation

The use of intraarterial lidocaine before and after liver directed chemoembolization and embolization procedures has demonstrated safety and efficacy with respect to subjective pain both during and after therapy, resulting in a significant decrease in narcotic requirements when undergoing embolization.^22^, ^23^, ^24^, ^25^ Due to the innervation and arterial anatomy of the liver, the intraarterial administration of local anesthetic results in permeation into the subcapsular (and viscerally innervated) portion of the liver and exposure of anesthetic to the liver parenchyma, as well as the segmental portal triads and hepatic veins.

The adoption of these techniques when performing the increasingly popular embolization/ablation combined procedures (commonly referred to as ‘double hit’ therapy),^22^, ^23^, ^24^, ^25^ in our experience, has resulted in decreased overall pain during and after the ablation segment of the procedure. Slow infusion of 50 ​mg lidocaine diluted into 50 ​cc of normal saline, administered in the target vascular bed over 2–4 ​min, mitigates the potential for pain during the initial infusion thought to be due to the acidic/low pH of lidocaine at room temperature.^26^

## Overview of sedation/anesthesia

Pre-procedural preparation following informed consent involves the standard protocol for use of moderate sedation, with appropriate intravenous sedation medication in addition to nil per os (NPO) at minimum 8 ​h before procedure. Standard intravenous access and appropriate resuscitation protocols should be applied, including the potential use of reversal agents. Imaging and confirmation of visualization of both target lesion and target regional block/anesthesia compartment should be confirmed by either ultrasound (preferred) or CT guidance prior to preparing/sterilizing the surgical field.

Institutional moderate sedation protocols may be implemented and generally initiated in a gradual fashion. We approach anxiolysis, sedation, and analgesia in the following phases:

### Phase 1: preprocedural optimization

Oral or IV diphenylhydramine (25–50 ​mg PO or IV) and oral benzodiazepine (such as 1–2 ​mg lorazepam sublingual), upon arrival in the procedure suite provides anxiolysis, further decreasing ‘catch up boluses’ of opioids, thereby minimizing the downregulatory effects on centrally mediated drivers of respiration.

### Phase 2: initial IV sedation

A combination of short acting IV administered opioids and benzodiazepines are used to achieve moderate sedation, without the need for airway assistance. Active monitoring should be maintained throughout the course of the procedure (including oxygen saturation, cardiac monitoring, and capnography if available). Airway assistance and resuscitation equipment should be available if needed.

### Phase 3: nerve/regional block

Following imaging localization, preparation of the sterile field, and administration of local anesthetic, image guided localization of target anatomy is achieved and confirmed (Table 1).

**Table 1:** Overview of block techniques.

Regional Technique	Anatomical Access	Modality	Medication	Application	
Splanchnic	Posterior T11	CT or Fluoro	3cc 1% Lidocaine+epi 8cc 0.25% bupivicaine	General Hepatic Ablation	
Celiac Plexus	Celiac Axis origin	CT or Ultrasound	3cc 1% Lidocaine+epi 10 ​cc 0.25% bupivicaine	General Hepatic Ablation	
Perihilar	Periportal	CT or Ultrasound	3cc 1% lidocaine+epi 15 ​cc 0.25% bupivicaine	Lobar intraparenchymal lesions	
Hydrodissection with anesthesia	Peritoneal Cavity	CT or ultrasound	10 ​cc 1% lidocaine per 250 ​cc N/S or D5W	Pericapsular lesions requiring hydrodissection	
Phrenic Nerve Block	Anterior Surface of Anterior Scalene Muscle	Ultrasound	3cc 0.25% bupivacaine	Subdiagphragmatic and subcapsular lesion	
Intercostal/Paravertebral Nerve Block	Anterior to Transverse Process T7-T9 (range T6-T10), inferior aspect to costal groove	Ultrasound or CT	3-5cc 0.5% ropivacaine or 0.25% Marcaine per level	Subphrenic lesions, unable to visualize portal vein, or celiac access	
Intraarterial	Anatomical segment	Angiography	10 ​mg lidocaine diluted into 50 ​cc N/S	During combined embo/ablation	

### Phase 4: ablation

Prior to initiation of ablation, a secondary supplemental bolus of IV sedation and analgesia is administered with anticipation of transient increased demand during the ablation cycle.

### Phase 5: post ablation

Post-ablation recovery includes standardized protocols following administration of moderate sedation, with a transition to oral analgesics prior to discharge (typically 1–2 ​h post procedure). If required, low dose oral analgesic/opioid can be provided to the patient upon discharge (eg acetaminophen/codeine, or ASA/caffeine/butalbital) for up to 48 ​h post discharge.

## Conclusion

The incorporation of nerve block and regional anesthesia techniques in hepatic ablation procedures requires an understanding of the embryologic and morphologic features of the liver. Armed with this knowledge, the use and practical application of these techniques becomes self-evident:

1) Improved pre-, intra-, and post-procedural comfort
2) Decreased requirement of hospital resources
3) Faster recovery and discharge
4) Decreased complications associated with overdose of sedation
5) Decrease infection risk associated with Aerosol Generating Medical Procedures (AGMP) such as general anesthesia and airway management
6) Overall procedural room efficiency
7) Increased treatment intensity (larger lesions, use of microwave, and ability to perform ablation and embolization procedures during longer sessions)

## Declaration of competing interest

The authors declare that there is no conflict of interest for the manuscript.
